# Research on storage stability differences between ceftriaxone sodium products

**DOI:** 10.1038/s41598-023-48410-z

**Published:** 2023-11-28

**Authors:** Shuye Qi, Xiaomeng Chong, Shangchen Yao, Baoming Ning, Changqin Hu

**Affiliations:** National Institutes for Food and Drug Control (NIFDC), Beijing, China

**Keywords:** Chemistry, Optics and photonics

## Abstract

The conditions and mechanisms leading to stability differences between ceftriaxone sodium products were examined to ensure drug quality and efficacy. We used a combination of powder X-ray diffraction and thermogravimetric analysis to examine the differences between preparations for injection from different pharmaceutical processes to elucidate the changed processes by exposing samples to different humidity and high-temperature conditions. Water loss or absorption due to varying environmental humidity levels did not adversely affect the crystal structure, but could lead to the reversible redistribution of hepta-hydrate in the unit cell of generic products, causing its stability change. The irreversible distribution of hydrate may occur when generic drugs stored at 25 °C, whereas the brand-name products remained stable at 40 °C. Therefore, generic ceftriaxone sodium and its powder preparations would be acceptable by better controlled sealing and storing under cool conditions during storage period to meet the efficacy and stability.

## Introduction

Ceftriaxone sodium (CTX·3.5H_2_O), a crystalline powder, is a third-generation cephalosporin that was developed by Roche Pharmaceuticals (Basel, Switzerland) in the 1980s^1^. In 1991, ceftriaxone sodium and various generic preparations derived from it were successfully developed in China. However, the clinical effects of generic ceftriaxone sodium for injection were questioned by clinicians^2,3^. Compared with brand-name products, generic ones probably had a delayed onset time for critically ill patients, and interacted more easily with volatile components such as antioxidants in rubber stopper, which led to drug turbidity and even allergic reactions^4,5^. To clarify the quality differences between generic drugs and brand-name ones, many investigational efforts have been conducted. In our previous studies, we found that the insufficient salt formation observed for generic products may be the main reason for the slower curative effect^6^, and the easily isomerized triazine ring structure of the ceftriaxone sodium C3 side chain was shown to have a strong effect on the adsorption of antioxidant butylated hydroxytoluene (BHT)^1^, which might lead to reduced efficacy, increased risk of clinical failure, and/or increased risk of emergence of resistant isolates for the generics.

Based on the improvement of pharmaceutical process, there were no significant differences in the physical and chemical laboratory tests between the new-produced generic products and the branded original ones for ceftriaxone sodium. However, their long-term ceftriaxone stability still needs more attention. Controlling water presence in formulation was shown critical, as ceftriaxone degraded in the presence of water content above 2.4% w/w^7^. This result indicated that water may affect the storage stability of ceftriaxone sodium products. In order to elucidate the affecting factors of storage process, the tests performed under different humidity and temperature conditions were conducted to simulate the real-world storage conditions of CTX·3.5H_2_O. Then, their changed micro-structure states were characterized by powder X-ray diffraction (PXRD), scanning electron microscopy (SEM) and thermogravimetric analysis (TGA) to understand the differences between generic and brand-name ones (Table 1).

**Table 1:** Certain words and their abbreviations.

Word	Abbreviation
Ceftriaxone	CTX
Ceftriaxone sodium	CTX·3.5H_2_O
Butylated hydroxytoluene	BHT
Powder X-ray diffraction	PXRD
The relative intensities of diffraction peak 3 and peak 4 in Fig. 1	I_4_/I_3_
Scanning electron microscopy	SEM
Thermogravimetric	TG
Thermogravimetric analysis	TGA
High-resolution TGA	hi-res TGA
Near-infrared spectroscopy	NIR
High performance liquid chromatography	HPLC
Chinese Pharmacopeia 2015	ChP2015

## Materials and methods

### Materials

Five batches of ceftriaxone sodium for injection, including three batches of generic preparations from Shanxi Pude Pharmaceutical Co., Ltd (batches 22190501, 22190502, and 22190503) and two batches of brand-name drugs from Roche Pharmaceuticals (batches B0467B03 and SH6550) were used. Drying, wetting, and high-temperature exposure were performed using these samples, as shown in Table 2.

**Table 2:** Test conditions.

Factor	Test number	Test conditions
Drying	Test 1-1	Samples in vials, with the rubber stopper removed, were stored in a glass dryer with allochroic silica gel for 0, 2, 4, 6, 16, or 33 d under cool and dark conditions
	Test 1-2	Samples in vials, with the rubber stopper removed, were stored at 2.0 kPa and 30 °C for 0, 2, or 8 h. Samples placed in a Petri dish were stored at 2.0 kPa and 30 °C for 6 h and recorded as 6 h (s), as shown in Fig. 6
Wetting	Test 2-1	Samples in vials, with the rubber stopper removed, were stored in a high-humidity environment under 60–80% relative humidity for 0, 5, or 16 d
High temperature	Test 3-1	Sealed samples were stored at 25 °C for 6 mo
	Test 3-2	Sealed samples were stored at 40 °C for 6 mo
	Test 3-3	Sealed samples were stored at 60 °C for 5 or 10 d
Long-term stability	Test 4	Sealed samples were stored at 40 °C and 75% relative humidity for 1, 3, or 6 mo

### PXRD

PXRD spectra were recorded using a Rigaku Smartlab diffractometer (Rigaku, Tokyo, Japan) with the following settings: 45 kV, 200 mA, and Cu Kα radiation (λ = 1.541862 Å). A D/teX Ultra 250 detector (Rigaku) was used in 1D mode. The samples were scanned at a rate of 8°/min in the 2θ range from 3° to 60° with steps of 0.01°. The incident Soller slit, incident slit, and the length of the limiting slit and receiving slit were 5.0°, 1/2°, 10.0 mm, and 20.0 mm, respectively. Some test powder was transferred onto a sample wafer and spread to cover the entire area of the wafer. The prepared test sample wafer was placed on the sample holder in the X-ray powder diffractometer (Table S1; Fig. S1).

### SEM

SEM images were obtained using a Phenom ProX desk-type SEM (Thermo Fisher Scientific, Waltham, USA) at 5.0 kV and 0.1 Pa, with sample surfaces coated by a thin platinum layer to avoid charging effects.

### TGA

Both standard TGA and high-resolution (hi-res) TGA were carried out using a TA TGA5500 instrument (TA Instruments; New Castle, DE, USA) over a temperature range of 30–300 °C, with heating rates of 5 and 30 °C/min, respectively. Especially, hi-res TGA was scanned using a resolution of 5 and sensitivity of 4 after equilibrating at 50 °C for 90 min. After weighing, each sample, limited to 2.5–3.0 mg, was transferred onto the sample tray and covered, and then placed into the corresponding platinum crucible for analysis.

### Near-infrared spectroscopy

Samples were scanned in vials using a Fourier transform near-infrared spectroscopy (NIR) integrating sphere (MPA, Bruker, Switzerland). A scan wavenumber range of 12,000–4000 cm^−1^ and a resolution of 8 cm^−1^ were used. Spectra were obtained by averaging the results of 32 scans, and three spectra of the same sample were averaged to give a mean spectrum. The optimized NIR model of water was built using partial least squares regression at 6993–5527 cm^−1^ and 4602–4227 cm^−1^ after processed by first derivative and vector normalization, and it was used to predict the water content and examine the respective changes.

### Serum protein binding rate

Solution A was a solution of Human Serum Albumin (SIGMA, Lot: SLBT3708, Assay ≥ 96%) (660 mg) in water (10 ml), and Solution B was a solution of the sample (16.3 mg) in water (25 ml). Solution A (1.0 ml) and Solution B (1.0 ml) were mixed to be the sample solution, taken to a water bath at 37 °C for 10 min and then centrifuged for 90 s at 25,000 rmp using Amicon Ultra-0.5 ml Centrifugal Filter Devices (MERCK) with an Ultracel-30 membrane. The blank solution was the mixed solution of water (1.0 ml) and Solution B (1.0 ml), prepared in the similarly as the sample solution. After preparation, both the blank and sample solutions were monitored by high performance liquid chromatography (HPLC) (Waters 2695–2996) with a PDA detector at 254 nm. The value of serum protein binding rate was calculated using the formula 1 − *A*_*sample*_/*A*_*blank*_, where A was the peak area of ceftriaxone in HPLC.

### Salt formation rate

The sodium ion content was measured using an iCE 3500 Atomic Absorption Spectroscopy (Thermo Fisher Scientific) instrument using the standard curve method (5 points: 1, 2, 3, 4 and 5 μg mL^−1^) at 589 nm using a Single Element Sodium Reference (National Center of Analysis and Testing for Nonferrous Metals and Electronic Materials, Lot: 191068-3, Assay = 1000 μg mL^−1^). The concentration of the sample solution was 40 μg mL^−1^. The salt formation rate was calculated based on the theoretical 2 sodium ions per formula unit (calc: 6.95%).

### Clarity measurement

The clarity of solutions was measured using a Hach 2100AN nephelometer and the second method (turbidity) described in the General Principle 0902 of Chinese Pharmacopeia 2015 (ChP2015). The turbidity value of the blank solution was measured first, followed by the measurement of the turbidity value of the sample solution. The concentration of the sample solution was 25 mg mL^−1^.

## Results and discussion

### Analysis of long-term stability tests

In order to clarify the differences in the storage of ceftriaxone sodium for injection produced by different pharmaceutical processes, long-term stability tests (Test 4 of Table 2) were carried out by detecting the serum protein binding rate, salt formation rate, rubber stopper compatibility, and corresponding diffraction patterns. Among these parameters, the protein binding rate and salt formation rate mainly affected the initial drug concentration in blood, and the rubber stopper compatibility could be used to reflect the stability of the product quality.

As shown in Table 3, there was no significant difference among the products at 0d in terms of protein binding rate and clarity, suggesting that the short-term stored generic products met the pharmaceutical quality of the branded original ones. However, there existed differences in clarity, protein binding rate and salt forming rate among samples after stored for 6 mo. According to the sample with poor stability performing higher protein binding rate, lower salt forming rate and worse clarity, the stability of these samples followed an order as generic product (batch 22190502) < generic products (batches 22190501 and 22190503) < brand-name products. Therefore, more attention should be paid to the long-term stability of generic ones, especially the batch 22190502.

**Table 3:** Critical quality attributes of the ceftriaxone sodium samples for injection.

Product classification	Batch no	Protein binding rate/%	Salt formation rate/%	Mean (SD) of compatibility tests of rubber stopper	
				Clarity (0 d)	Clarity (6 mo)
Chinese generic drugs	22190501	80.22	1.87	0.444 (0.025)	0.631 (0.059)
	22190502	78.18	1.83	0.400 (0.031)	0.909 (0.085)
	22190503	76.48	1.82	0.414 (0.016)	0.643 (0.091)
Chinese brand-name drugs	SH6550	77.05	1.83	0.404 (0.025)	0.478 (0.026)
Brand-name drugs	B0467B03	74.68	2.16	0.494 (0.035)	0.454 (0.031)

*SD* standard deviation (of a sample).

### Analysis of PXRD and SEM

**Figure 1:**
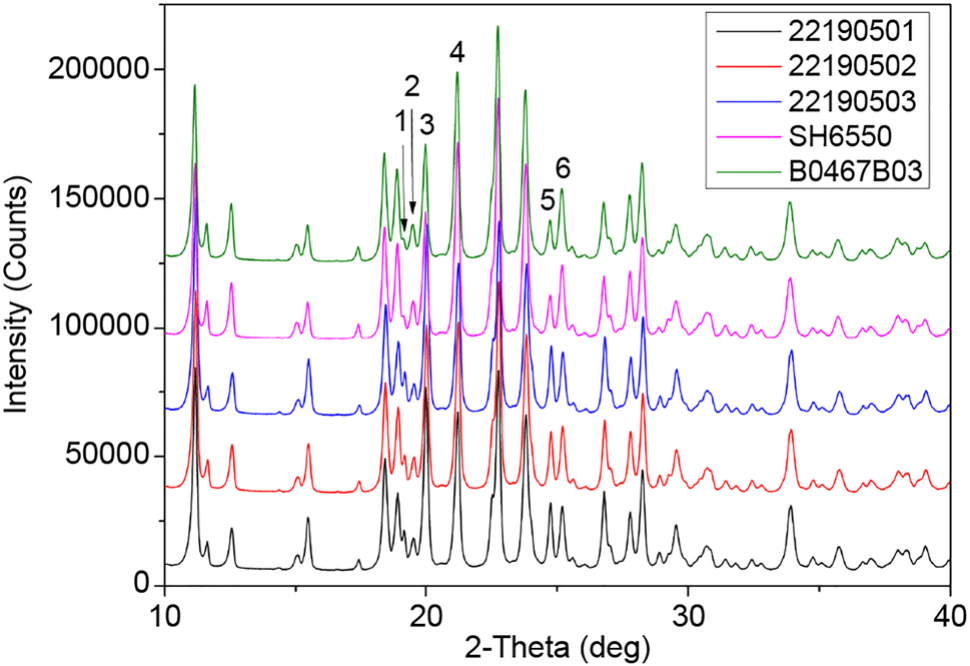
PXRD spectra of the as-received samples in the 10°–40° range, and numbers 1 ~ 6 were marked at peaks with varied intensities and widths in these diffraction spectra.

The PXRD spectra of all samples are shown in Fig. 1. The angular positions of the diffraction peaks were the same, which indicated that the geometry and size of the unit cell of these samples were similar. But some variations of intensities and widths of peaks were clearly observed in diffraction spectra of Fig. 1.

The difference in the peak intensities, mostly excluding preferential orientation effects through multiple measurements, was probably due to variances in the atomic arrangement in unit cells between generic and brand-name products. It is necessary to elucidate the molecular group characteristics of CTX·3.5H_2_O, and the relative intensities of diffraction peaks, especially I_4_/I_3_, may be signatures of atomic arrangement. The triazine ring structure of the ceftriaxone C3 side chain was easily isomerized, showing keto-enol tautomerism^8^, which suggest that the varied atom arrangements between ceftriaxone products probably occurred in the group at C-3.

**Figure 2:**
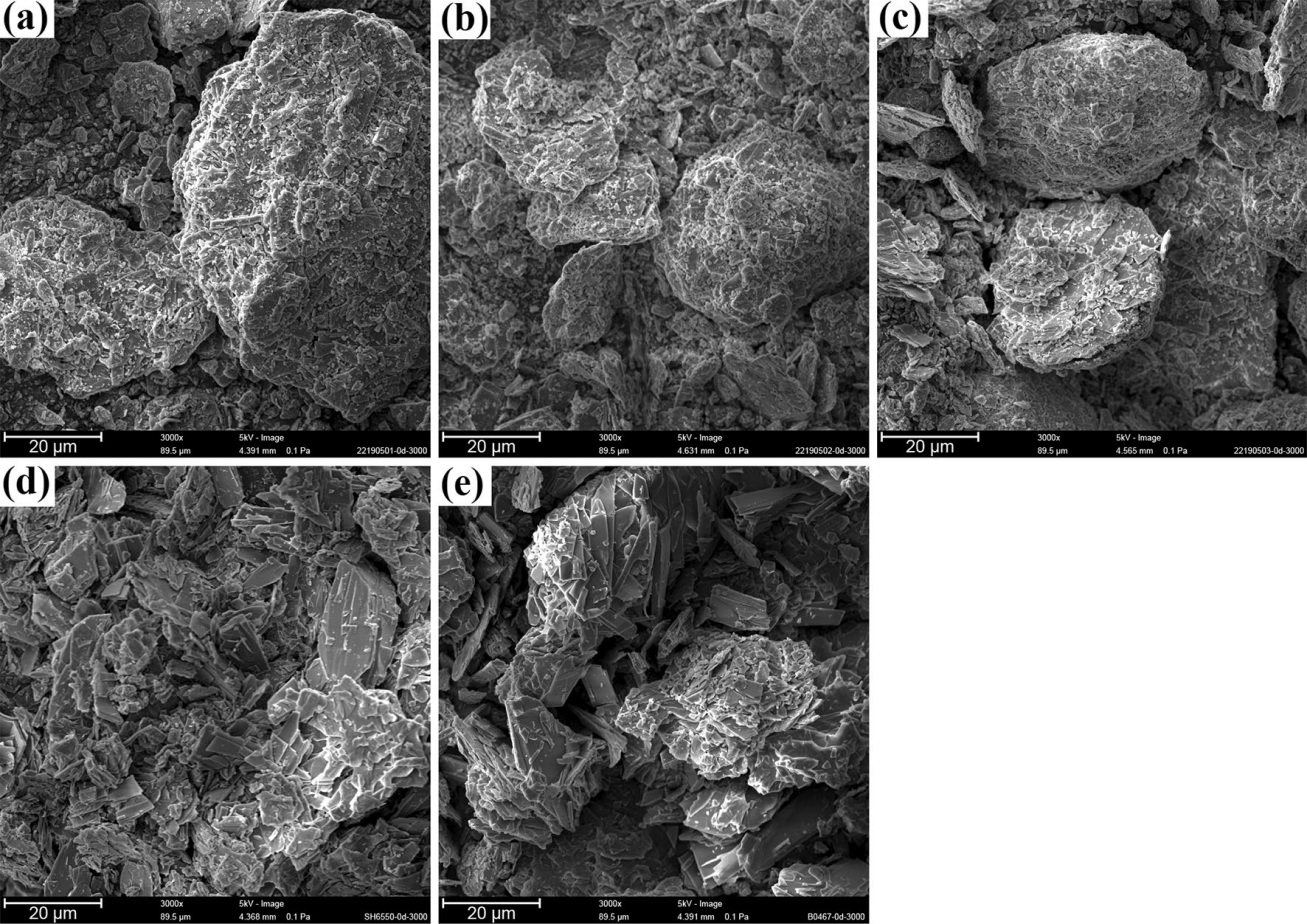
SEM images of (**a**) 22190501; (**b**) 22190502; (**c**) 22190503; (**d**) SH6550; and (**e**) B0467B03.

**Figure 3:**
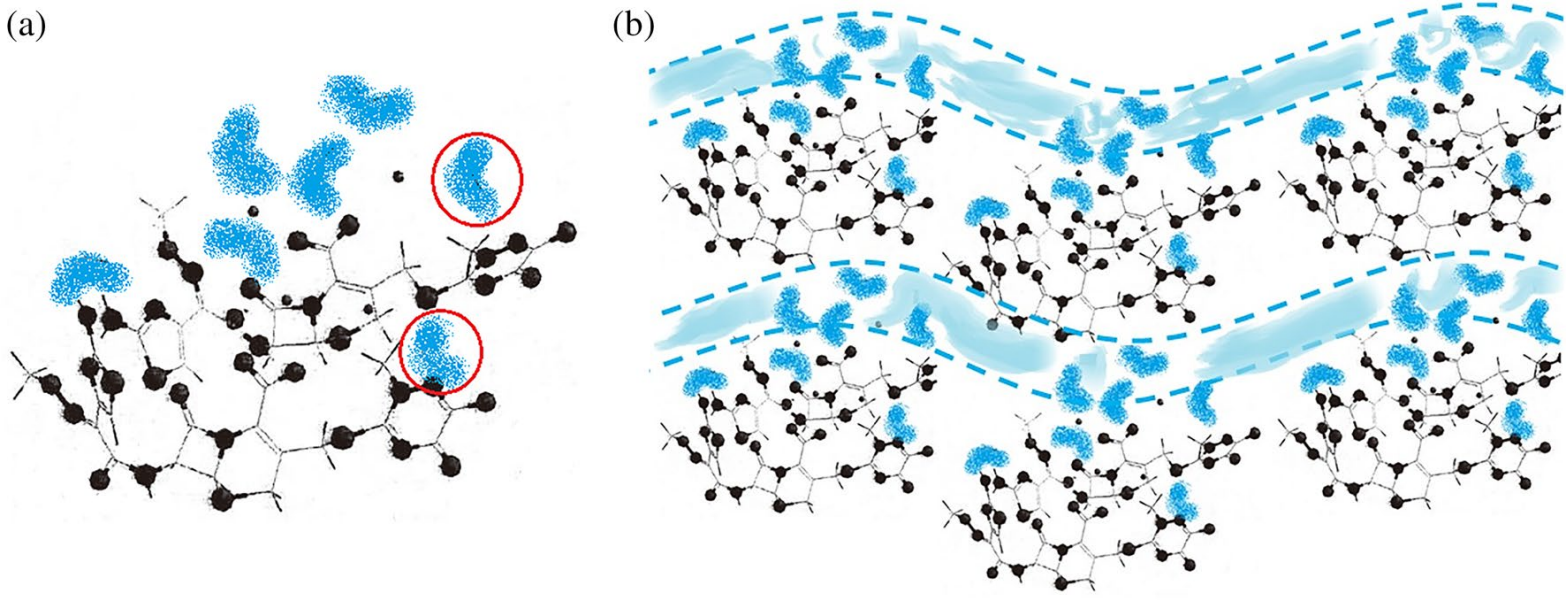
(**a**) Molecular stereo-structure of ceftriaxone sodium simulated using Cerius^2^. The two water-binding sites marked by the red circles are near the easily isomerized triazine ring structure of the ceftriaxone sodium C3-side chain. (**b**) Packing motif of CTX·3.5H_2_O. Blue markers indicate isolated water-binding sites in the unit cell, and light blue areas indicate channels of water molecules. One of the two sites marked by the red circles may be located in these channels, while the other may be isolated.

The peak broadening of generic samples could be related to crystallite size reduction shown as the SEM results of Fig. 2. According to the SEM results, all samples consisted of flake crystals, which prevented the use of single X-ray diffraction to determine the crystal structure of CTX·3.5H_2_O. One group^9^ modeled the molecular stereo-structure of ceftriaxone sodium using Cerius^2^ software and reported that 2 ceftriaxone sodium and 7 water molecules constitute a crystal unit cell (Fig. 3a), where the molecules were connected by ionic, hydrogen, and other bonds.

### Analysis of TGA

**Figure 4:**
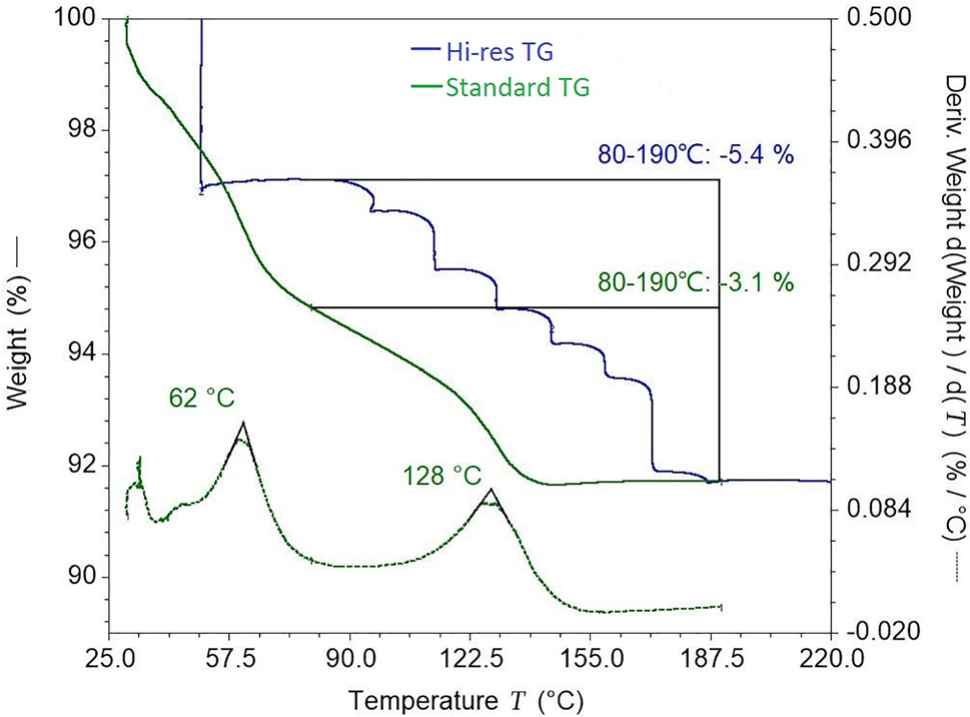
Typical TGA curves (standard and high-resolution TGA) of B0467B03 (subtype II).

The representative curve from standard TGA is shown in Fig. 4. The weight loss from 30 to 200 °C was around 9.2%, which was consistent with the water content determined using Fisher’s method (as described in General Principle 0832 of ChP2015) and also close to the theoretical 3.5 water molecules per formula unit (calc: 9.52%). This suggests that the weight loss of CTX·3.5H_2_O from 30 to 190 °C was related to the dehydration of hydrate, and coordinated water was completely removed only at temperatures above 190 °C. Furthermore, there were two peaks in the derivative line of the standard TGA curve, which indicated that CTX·3.5H_2_O was dehydrated in two steps, with the loss of 2.5H_2_O (about 6.1%) in the first step from 30 to 80 ℃ with a peak temperature of 62 ℃ (CTX·3.5H_2_O → CTX·H_2_O + 2.5H_2_O) and that of H_2_O (about 3.1%) in the second step from 80 to 190 ℃ with a peak temperature of 128 ℃ (CTX·H_2_O → CTX + H_2_O). These results are consistent with previous findings^10^. Thus, we cite their kinetic parameters, such as the dehydration activation energy *E* obtained by the Coats–Redfern method, to help understand the non-isothermal dehydration kinetics of CTX·3.5H_2_O. The *E* values for the first and second steps were 95.3 and 151.3 kJ/mol, respectively. According to a classification of hydrates^11^, the hydrate from the first step (CTX·3.5H_2_O) was mostly a Class 1 hydrate with low dehydration *E*, which is easy to rehydrate to the original hydrate form, even when it is well dried. The hydrate from the second step (CTX·H_2_O) was probably a Class 3 hydrate with high dehydration *E*, which requires energy to both remove water molecules and rearrange the crystal. They demonstrated that water molecules are arranged one-dimensionally like channel hydrates in Class 1, and like isolated hydrates in Class 3^11^. Therefore, the design of the packing motif of CTX·3.5H_2_O shown in Fig. 3b is considered reasonable, and the crystal transformation might be mainly related to water molecules at the isolated sites.

To increase the transition resolution and enhance signature analysis capability, hi-res TGA was used to distinguish the slight differences in the dehydration behavior of CTX·3.5H_2_O. The hi-res TGA curves shown in Fig. 4 were scanned at 30 °C/min after equilibrating at 50 °C for 90 min. The total weight loss of batch B0467B03 was around 9.2%, which is equal to its loss determined by standard TGA. In particular, the curve did not show continuous weight loss, but rather seven steps between 80 and 190 °C. This is consistent with the typical dehydration behavior of stoichiometric hydrates. According to the seven dehydration steps with different weight losses, it was verified that seven water-binding sites were present in the unit cell of CTX·3.5H_2_O, shown in Fig. 3a, and their binding ability differed. Combined with the PXRD results, the two binding sites near the C-3 group (shown in Fig. 3) might be the source of the different properties between ceftriaxone products.

### Sensitivity of ceftriaxone sodium to ambient humidity and temperature

#### Effect of humidity

**Figure 5:**
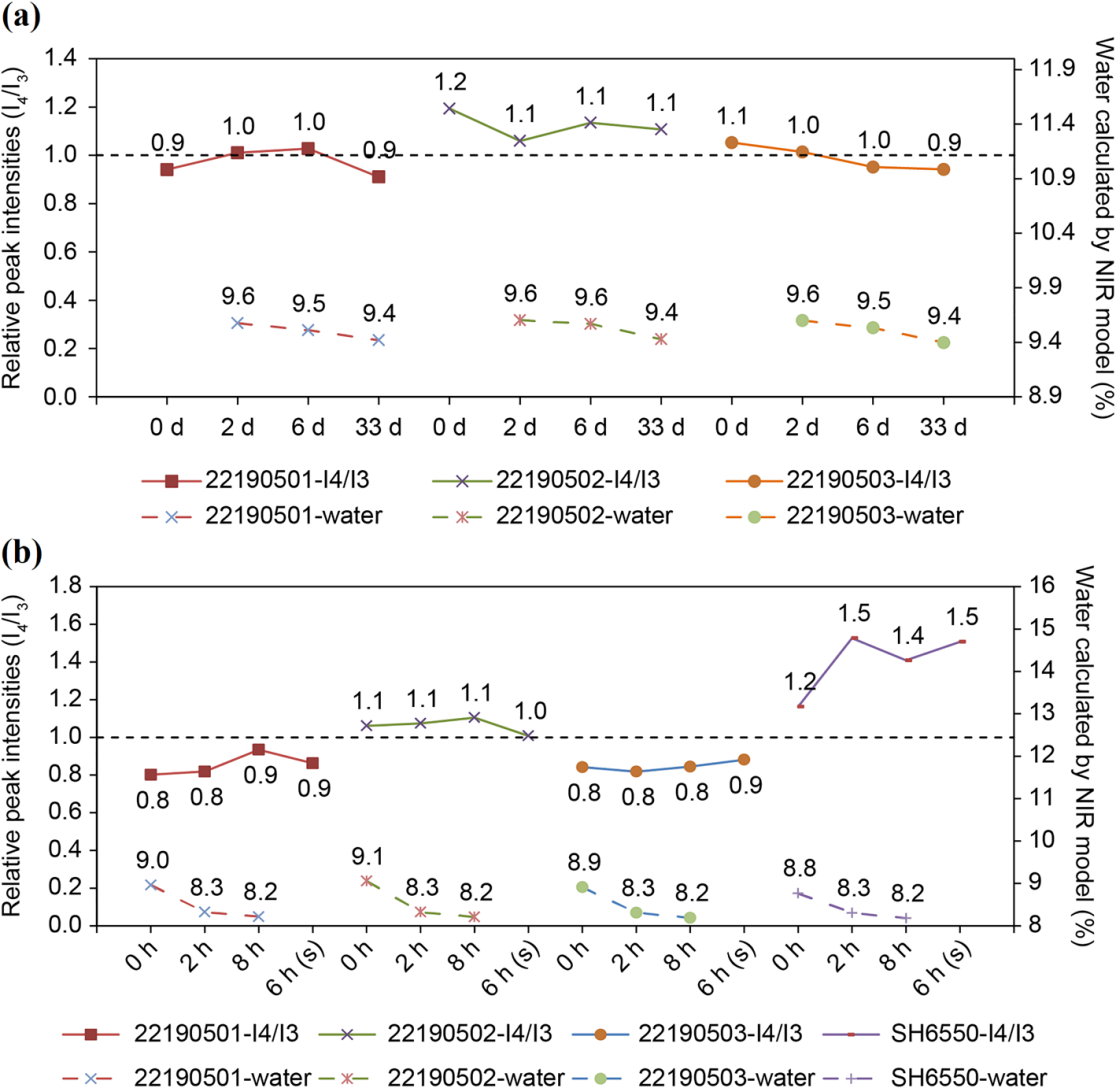
Comparison of PXRD results and water contents of samples used in the drying tests (Table 1). (**a**) Test 1-1. (**b**) Test 1-2. The results of PXRD are described by relative peak intensities I_4_/I_3_, and the water content was predicted using NIR models.

The dehydration and rehydration behaviors of samples stored under controlled-humidity conditions were characterized using hi-res TGA and PXRD. First, samples were treated according to the drying conditions used in Tests 1-1 and 1-2 (Table 2). Based on the results of PXRD (Fig. 5), the changes of microstructural parameters from I_4_/I_3_ > 1 to I_4_/I_3_ < 1 only occurred in generic samples. Combined with the results of hi-res TGA (Fig. 7), the generic products changed the dehydration characteristics in steps 6 and 7, and the sample of batch 22190502 returned to a new balanced state after drying for 16 d while the other samples of batches 22190501 and 22190503 returned to the original form. This suggests that the bound water molecules might be rearranged in the CTX·3.5H_2_O crystal and reached equilibrium after long-term drying. However, the brand-name products (batches B0467B03 and SH6550) always maintained their crystal states, indicating that the water-binding ability of generic products was weaker than that of brand-name ones, and it was necessary to distinguish these two product forms.

**Figure 6:**
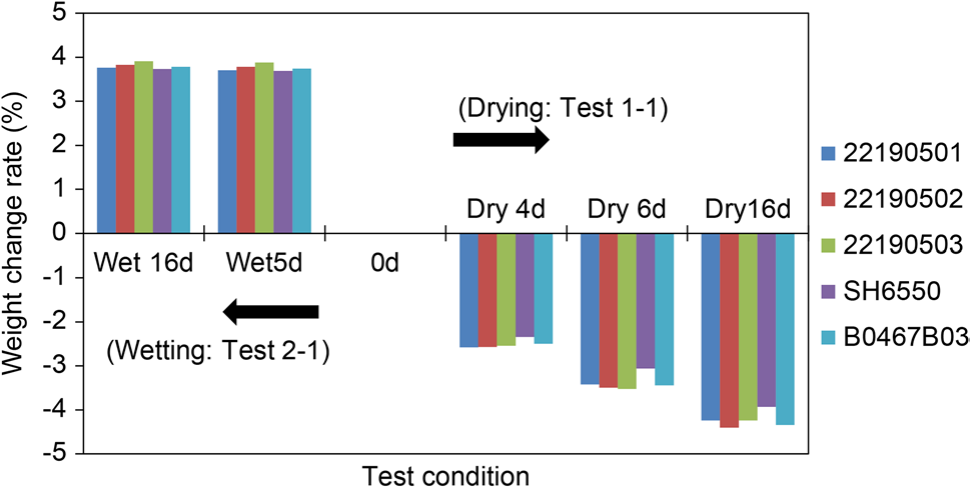
Weight change rates after Test 1-1 and Test 2-1 treatments (Table 1).

**Figure 7:**
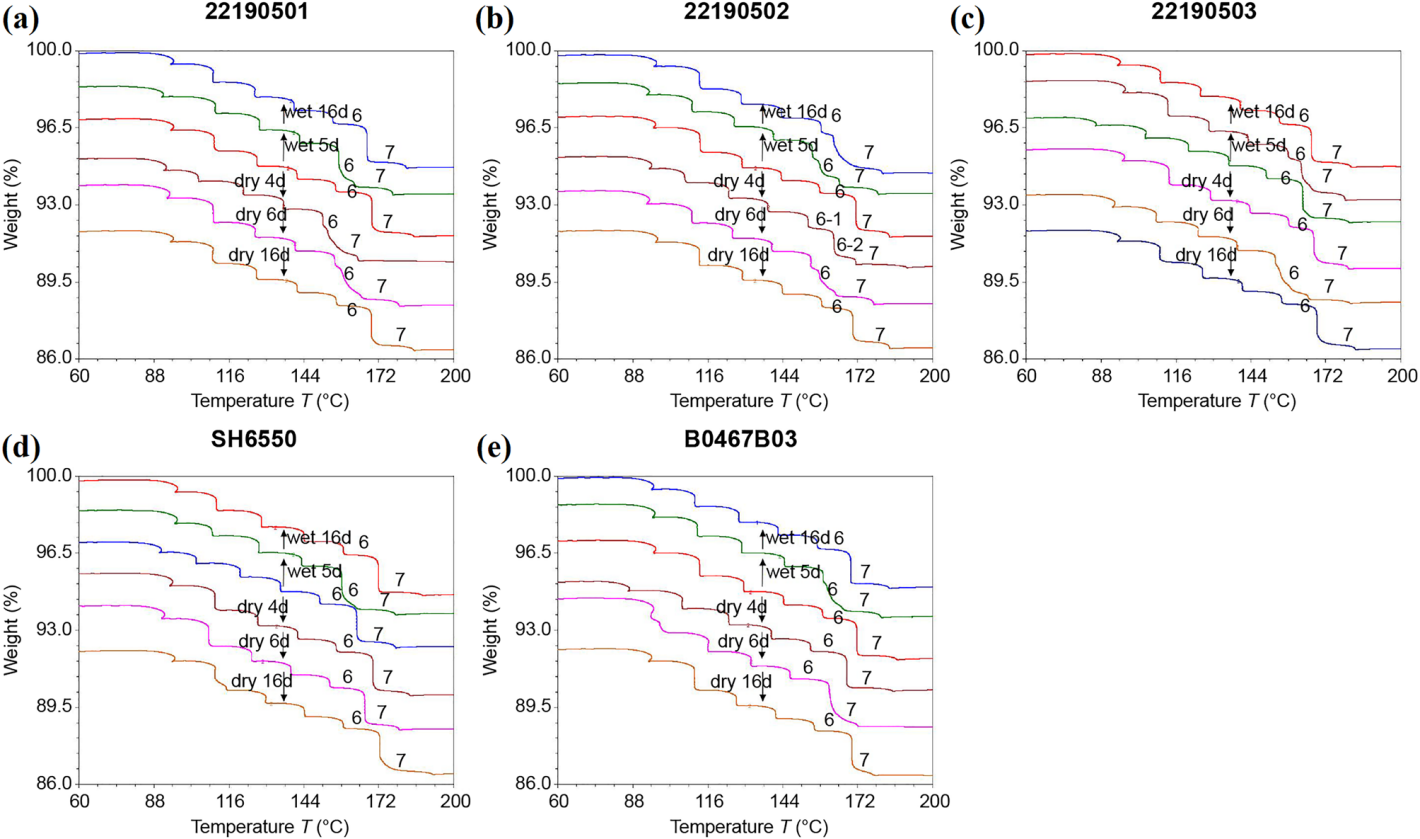
TGA curves after Test 1-1 (drying for 4, 6, and 16 d) and Test 2-1 (wetting for 5 and 16 d; Table 1) treatments.

Furthermore, the samples were treated according to Test 2-1, and the results are shown in Figs. 6 and 7. Figure 6 shows that the weight increase rates of samples under “wet 5 d” were close to those under “wet 16 d”, which indicates that the water absorption capacity of ceftriaxone sodium was limited in the high-humidity environment. After wetting for 16 d in a high-humidity environment, only the TG curve of generic sample (batch 22190502) didn’t return to a balanced state, and the microstructural parameters of generic samples (batches 22190501 and 22190503) changed from I_4_/I_3_ < 1 to I_4_/I_3_ > 1.

The drying and wetting tests showed that the hydrate classification of Class 1 and Class 3 for CTX·3.5H_2_O was reasonable, and the loss or gain of water because of differences in environmental humidity during storage did not adversely affect the crystal structure of CTX·3.5H_2_O, but could lead to the redistribution of water in the unit cell, resulting in a change in the generic products.

#### Effect of temperature

**Figure 8:**
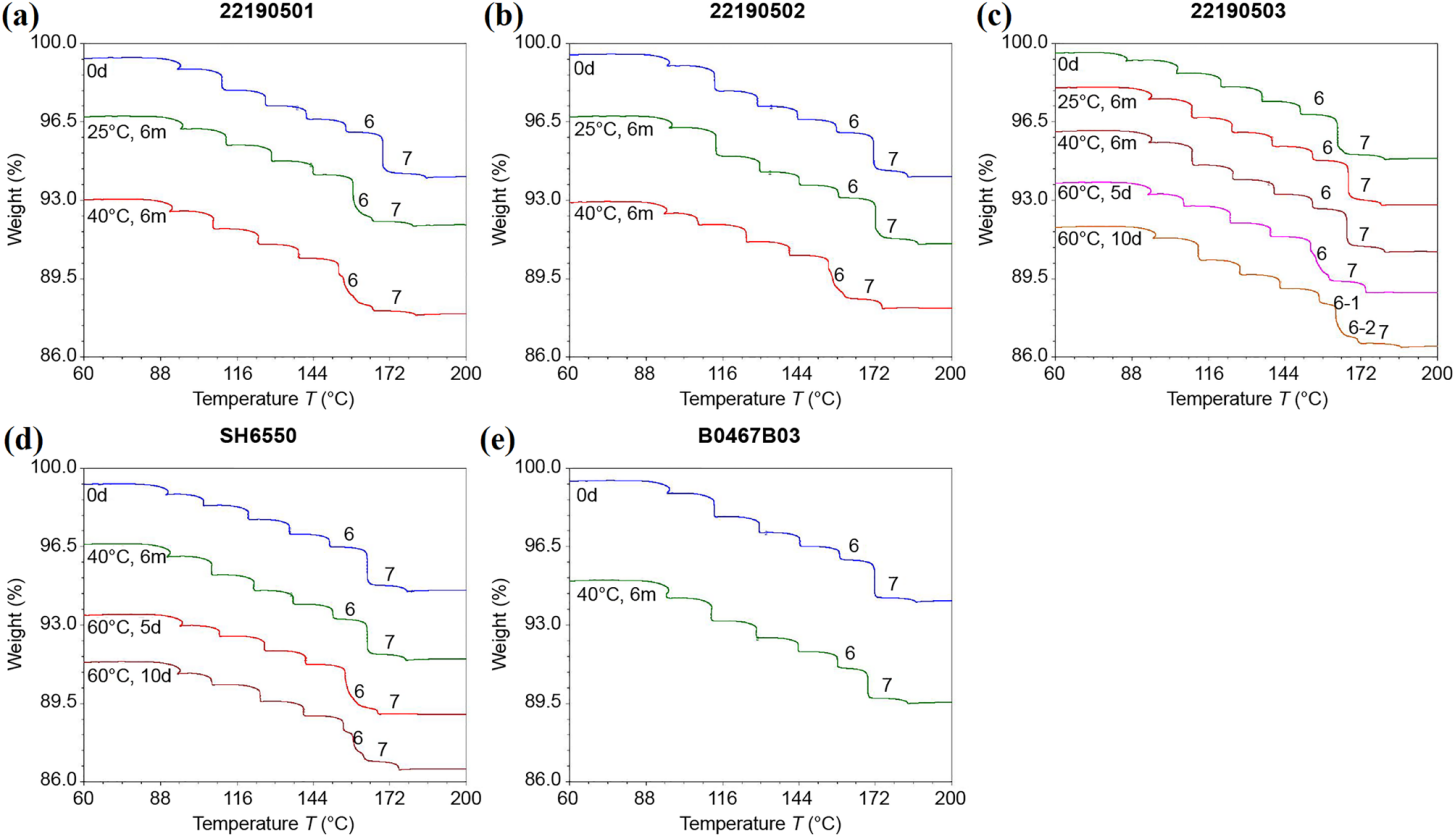
TG curves of all initial samples after Test 3 treatment (Table 1).

The effects of temperature on sample stability during sealed storage were investigated under the conditions of Test 3, and the results are shown in Fig. 8. In the brand-name products (batches B0467B03 and SH6550), the initial state of hydrate was maintained over a long period when stored at or below 40 °C. However, a different behavior was observed for the generic products (batches 22190501, 22190502, and 22190503). The stability changes of batches 22190501, 22190502, and 22190503 occurred at 25, 40, and > 40 °C, respectively. Thus, it was verified the stability of hydrate was higher for the brand-name products compared to the generic products.

In addition, the TG characteristics shown in Fig. 8 did not return to their initial state after storage for 6 mo at 40 °C or after several days (5 and 10 d) at 60 °C, suggesting that the water redistribution caused by high-temperature exposure was irreversible. This may be associated with the destruction or transformation of the crystal structure at high temperatures.

## Conclusions

After comparing the stability of the samples in different storage conditions, the stability of hydrate was higher for the brand-name products compared to the generic products. The generic samples was a metastable form with changing water distribution. According to the reversible redistribution of hydrate in the unit cell under different ambient humidity and low temperature (< 25 °C), CTX·3.5H_2_O and its powder preparations should be packaged protected against humidity and kept in cold storage.

However, according to the comparison between protein binding rate and rubber stopper compatibility (0d), the generic products were also acceptable, but sealing package and cool condition during the storage period must be provided to ensure the stability of the products.

### Supplementary Information

Supplementary Information.

## Data Availability

The datasets generated in this study are available upon request from the corresponding author.
