# Supplementary material for: Research on storage stability differences between ceftriaxone sodium products
Source: Sci Rep. 2023 Nov 28;13:20996. doi: 10.1038/s41598-023-48410-z (PMC10684491; doi:10.1038/s41598-023-48410-z)
Supplement: Supplementary file 1 — Supplementary Information. [file 41598_2023_48410_MOESM1_ESM.docx]

**SUPPLEMENTS**

As shown in Figure S1 and Table S1, each sample was measured thrice in the X-ray powder diffractometer, and their similar spectra were used to prove the negligible influence of preferred orientation on peak intensities.







**Figure S1.** PXRD spectra of the samples (batches 22190501 and 22190502) in the 18–26° range.

**Table S1.** PXRD intensities of the samples (batches 22190501 and 22190502) at the 3^rd^ and 4^th^ peaks.

| Batch no.  Intensity | 22190501 | | | 22190502 | | |
| --- | --- | --- | --- | --- | --- | --- |
|  | 1 | 2 | 3 | 1 | 2 | 3 |
| I_3_ | 76836 | 74762 | 75516 | 67139 | 66737 | 66947 |
| I_4_ | 67122 | 66291 | 66644 | 73766 | 74009 | 76315 |
| I_4_/I_3_ | 0.87 | 0.89 | 0.88 | 1.10 | 1.11 | 1.14 |
